# Preparation for the next pandemic: challenges in strengthening surveillance

**DOI:** 10.1080/22221751.2023.2240441

**Published:** 2023-08-31

**Authors:** Kelvin Hei-Yeung Chiu, Siddharth Sridhar, Kwok-Yung Yuen

**Affiliations:** a Department of Microbiology, Queen Mary Hospital, Hong Kong Special Administrative Region, People's Republic of China; b State Key Laboratory for Emerging Infectious Diseases, Carol Yu Centre for Infection, Department of Microbiology, Li Ka Shing Faculty of Medicine, The University of Hong Kong, Hong Kong Special Administrative Region, People's Republic of China; c Department of Microbiology, School of Clinical Medicine, Li Ka Shing Faculty of Medicine, The University of Hong Kong, Hong Kong Special Administrative Region, People's Republic of China; d Department of Infectious Disease and Microbiology, The University of Hong Kong-Shenzhen Hospital, Shenzhen, People’s Republic of China; e Centre for Virology, Vaccinology and Therapeutics, Hong Kong Science and Technology Park, Hong Kong Special Administrative Region, People's Republic of China

**Keywords:** COVID-19, pandemic, surveillance, zoonotic, outbreak

## Abstract

The devastating Coronavirus Disease 2019 (COVID-19) pandemic indicates that early detection of candidates with pandemic potential is vital. However, comprehensive metagenomic sequencing of the total microbiome is not practical due to the astronomical and rapidly evolving numbers and species of micro-organisms. Analysis of previous pandemics suggests that an increase in human–animal interactions, changes in animal and arthropod distribution due to climate change and deforestation, continuous mutations and interspecies jumping of RNA viruses, and frequent travels are important factors driving pandemic emergence. Besides measures mitigating these factors, surveillance at human–animal interfaces targeting animals with unusual tolerance to viral infections, sick heathcare workers, and workers at high biosafety level laboratories is crucial. Surveillance of sick travellers is important when alerted by an early warning system of a suspected outbreak due to unknown agents. These samples should be screened by multiplex nucleic acid amplification and subsequent unbiased next-generation sequencing. Novel viruses should be isolated in routine cell cultures, complemented by organoid cultures, and then tested in animal models for interspecies transmission potential. Potential agents are candidates for designing rapid diagnostics, therapeutics, and vaccines. For early detection of outbreaks, there are advantages in using event-based surveillance and artificial intelligence (AI), but high background noise and censorship are possible drawbacks. These systems are likely useful if they channel reliable information from frontline healthcare or veterinary workers and large international gatherings. Furthermore, sufficient regulation of high biosafety level laboratories, and stockpiling of broad spectrum antiviral drugs, vaccines, and personal protective equipment are indicated for pandemic preparedness.

## Introduction

The pandemic of Coronavirus disease 2019 (COVID-19), caused by severe acute respiratory syndrome coronavirus 2 (SARS-CoV-2), has affected more than 768 million people with 6.9 million deaths as of 19 June 2023 [1]. Previous studies have estimated the economic loss from COVID-19 to be at least 16 trillion USD, taking into account premature deaths, long-term physical and mental health impairment, and loss of workforce secondary to social distancing measures [2]. Given the devastating consequences of pandemics, earlier detection of candidates with pandemic potential is vital.

## Difficulties in total microbiome sequencing

Surveillance is an important component of pandemic preparedness and can be classified into indicator-based surveillance and event-based surveillance [3]. Indicator-based surveillance refers to traditional surveillance using notifications based on the clinical information provided by the health care system and laboratory confirmation of the pathogen. In order to predict candidates for future pandemics, indicator-based surveillance through microbial surveillance of animals by high throughput sequencing technology has been widely used since zoonotic spillover constitutes 75% of all emerging infectious diseases [4]. Theoretically, comprehensive metagenomic sequencing for detection and characterization of the total microbiome (viromes, bacteriomes, fungiomes, and parasitiomes) in air, water, soil, plants, animals, and humans can detect all potential candidates. An initiative has been put forward to understand complete viromes. The Global Virome Project (GVP), an international collaborative research initiative, has been launched with its goal of identifying and preventing future outbreaks by large-scale collection and sequencing of unknown viruses of wildlife in biodiverse regions that are hotspots for emerging infections [5]. However, even this well-conceived approach may not be able to capture potential candidates for the next pandemic agent, given the astronomical and rapidly evolving numbers and species of micro-organisms in the ocean, soil, and atmosphere (Table 1) [6–9] and the number of animals and plants on Earth that can harbour these micro-organisms (the order of magnitude is approximately 10^18^ non-human animals, 10^12^ non-human mammals, 10^10^ birds, 10^9^ bats, more than 10^6^ Camelidae, 10^14^ mosquitoes, and 10^12^ trees). Studies have also estimated that the total number of unknown mammalian viruses with zoonotic potential is around 600,000 [5]. A recent virome characterization study performed in China identified only 65 mammal-infecting viruses in 1941 game animals, suggesting that far more sampling is required to cover enough of the virome in order to identify candidate viruses for future pandemics [10]. Furthermore, biodiversity-based prediction is unlikely to compensate for the rapid evolution of RNA viruses unless surveillance is performed continuously. Therefore, a more feasible and cost-effective approach is required for surveillance.

**Table 1.** Number of micro-organisms in the environment according to literature.

Microorganisms	Soil (g^−1^)	Ocean (L^−1^)	Atmosphere (m^−3^)
Bacteria	10^7^–10^9^ [6]	10^9^ [7]	10^2^–10^6^ CFU [8]
Viruses	10^3^–10^9^ [9]	10^10^ [7]	10^5^^,^^a^ [8]
Fungi	10^5^–10^6^ [6]	Data not available	10^2^–10^3^ spores [8]

^a^Virus-like particles detected by fluorescence microscopy.

## Factors associated with the development of pandemics

Recorded pandemics have been due to both bacterial and viral infections (Table 2) [11–17]. Pandemics have occurred regularly throughout recorded history, with culprits of pandemics shifting from bacteria to RNA viruses because plague and cholera are preventable with sanitation and rodent control measures, and are also highly treatable with antibiotics. Furthermore, bacterial pathogens acquired from the environment such as *Burkholderia pseudomallei* and *Legionella pneumophila* are less likely to cause pandemics, except those that can be transmitted from person to person, such as *Vibrio cholerae*. While bacterial pandemics were mostly attributed to poor sanitation, recent pandemics due to respiratory viruses were multifactorial, including the ability of RNA viruses to mutate, recombine and reassort, viral shedding from asymptomatic or mildly symptomatic individuals, and an increase in human–animal interactions.

**Table 2.** Summary of significant epidemics and pandemics occurred in human history.

Pathogens	Epidemics/pandemics	Years	Estimated number of death	Details	Major reason(s) for pandemic development	Possible zoonotic origin of pandemics
*Yersinia pestis*	Plague of Justinian	541–543	100 million	Based on isolation of microbial DNA in dental pulp specimens	Poor environmental hygiene	Rodents
	Black Death	1347–1352	200 million	Followed by multiple waves in Europe		
	Third plague pandemic	1855–1959	12 million	Originated from Yunnan in China, then spread to Hong Kong in 1894 Discovery of *Yersinia pestis* as the culprit of plague		
*Vibrio cholerae* Classical biotype	Six pandemic of cholera	1817–1824 1826–1837 1846–1860 1863–1875 1881–1896 1899–1923	Unknown	Originated from India then spread via trade route worldwide	Poor environmental hygiene, Increased human travel	Potential element of transmission through consumption of aquatic animals
*Vibrio cholerae* El Tor biotype	Seventh pandemic of cholera	1961–now	Unknown	Originated from Indonesia		
Poliovirus	Polio epidemics	1900–1950s	More than 16,000	Spreading around the world, with major epidemics in the United States in 1916 and 1952. Situation improves after introduction of polio vaccines	Delayed in exposure due to improved sanitation, infection in older children leading to more severe presentation	Nil
Influenza A/H3N8	Russian flu	1889–1895	1 million	Predicted to be H3N8 based on serological and epidemiological data	Virus ability for gene reassortment, Increase in human travel	Unknown
Influenza A/H1N1	Spanish flu	1918–1919	50 million	Pandemic started in United States, then spread worldwide	Virus ability of gene reassortment and zoonotic spillover, Increase in human travel and poor hygienic condition due to World War I, Extreme weather	Suspected bird and pig
Influenza A/H2N2	Asian flu	1957–1959	1–2 million	Derived from 1918 virus by acquisition of three new avian gene segments (HA, NA, and PB1 polymerase) by reassortment [15]	Virus ability of gene reassortment, Increase in human travel	Suspected bird and pig
Influenza A/H3N2	Hong Kong flu	1968–1970	0.5–2 million	Antigenic shift from A/H2N2. Avian origin of PB1 leads to increase in replication and transmissibility, with new HA evading pre-existing immunity in human [16].		Suspected bird and pig
Influenza A/H1N1	Swine flu	2009	Laboratory confirmed: 18631. Estimated between 148,000 and 249,000 [36]	Triple gene reassortment originating from human A/H3N2 (PB1 polymerase), avian (PB2, and PA polymerase), and swine (H1, N1, NP, Matrix, and non-structural protein) influenza viruses [17]		Potential swine origin. Triple reassortment from human, avian, and swine influenza virus
Human immunodeficiency virus (HIV)	AIDS	1981–now	40 million	First case of AIDS reported in June 1981	Long incubation period (Infectious during asymptomatic phase)	African primates (multiple cross species transmission)
Severe acute respiratory syndrome coronavirus 2 (SARS-CoV-2)	COVID-19	2019–now	6.9 million	First case of COVID-19 described in December, 2019 in Wuhan, China Emergence of different variants during pandemics	Infectious during asymptomatic phase, RNA virus with airborne transmissibility and frequent mutations	Bat, possible unidentified intermediate host

It is just a matter of time before another pandemic emerges because collective alertness about pandemics declines as memories of the last pandemic fade. Novel infectious disease epidemics emerge from the interplay of human, environmental, and pathogen factors. Increasing interaction between wild animals, livestock and humans, together with large-scale international movements of humans and animals can rapidly spread infectious diseases to other parts of the world [18]. Travellers from epidemic centres have been a major cause of epidemics and pandemics. The first case of SARS-CoV-1 in 2003 in Hong Kong was a medical doctor who travelled from Guangzhou to Hong Kong and had preceding contact with patients suffering from severe acute respiratory syndrome. The first case of pandemic H1N1 in Hong Kong in 2009 was a Mexican national travelling to Hong Kong via Shanghai. The first identified family cluster of COVID-19 in 2019 was a Shenzhen family with members returning from a visit to Wuhan [19]. Overcrowding especially in cities promotes the rapid transmission of respiratory virus infections through viral shedding by airborne (inhalation), droplet (spray), and/or direct and indirect contact routes [20,21].

The increase in human and animal interactions as a result of the rising global population, the destruction of natural habitats, factory farming, and trafficking of exotic animals enhances the probability of zoonotic transmission, a necessary precursor to epidemics or pandemics. Outbreaks of poultry deaths in farms and markets were reported before or during outbreaks in humans for the 1997 avian influenza A H5N1. For both SARS-CoV-1 and SARS-CoV-2, human cases were traced to have a linkage with wild animals or wet markets [22–25]. Interestingly, the first case of H5N1 in Hong Kong in 1997 was a child with possible contact with sick chickens at a preschool [26]. In developing countries, overpopulation, encroachment into forest areas, and interactions with animals through animal contact or consumption have led to outbreaks of agents of viral haemorrhagic fever, including the Ebola virus and Lassa virus [27–29].

A warming world expands the geographical distribution of intermediate hosts such as mosquitos, hence increasing vector-borne outbreaks of dengue, Zika, chikungunya, and malaria [30]. With the widespread use of anti-malarial, insect repellent, and insecticides, pan-antimalarial-resistant *Plasmodium* species and insecticide-resistant mosquitos may cause severe outbreaks. Climate change and anthropogenic waste generation has also been linked to uptrends in urban rat populations with potential consequences for rodent-borne illness such as leptospirosis and rat hepatitis E [31]. Natural disasters and wars can disrupt public health infrastructure and degrade environmental hygiene which increases the transmission of pathogens such as *Yersinia pestis* and *Vibrio cholerae*, epidemic typhus during World War II as well as the severe pandemic of influenza A/H1N1 (Spanish flu) during World War I [32–35].

Demographic shifts will also impact the form that future pandemics take. Aging populations in many parts of the world imply a large susceptible immunosenescent population that might be particularly vulnerable to pandemic agents. For example, age is one of the most consistent risk factors for severe SARS-CoV-2 infection during the current COVID-19 pandemic [37]. On the other hand, the rising population in parts of Africa and the Indian subcontinent result in overcrowding and strains on public sanitation. These conditions, in turn, fuel the emergence and spread of novel pandemic agents, especially if combined with other factors detailed above.

The increasing number of pandemics related to RNA viruses such as coronaviruses and influenza viruses implies that they possess unique characteristics [38,39]. SARS-CoV-2 is infectious prior to the onset of symptoms and around 30%–60% of patients with COVID-19 are asymptomatic, posting difficulty in controlling the spread of SARS-CoV-2 in the community [40]. Furthermore, low fidelity of RNA-dependent RNA polymerase in emerging RNA respiratory viruses is associated with frequent mutations, hence leading to the emergence of different variants with increasing immune evasion and upper airway predilection as demonstrated in laboratory animals and human airway organoid cultures which augments their airborne transmissibility as in the case of SARS-CoV-2 [41–44]. Novel SARS-CoV-2 Omicron subvariants with immune evasiveness such as XBB.1.5 can emerge by recombination [45]. Frequent mutations resulting in antigenic drift and sometimes gene reassortment in virus with segmented genomes leading to antigenic shift allow the virus to re-infect previously immune populations, adapt to different host cell environments, and cause successful cross-species transmissions as exemplified by the human influenza A viruses [46,47], and avian influenza viruses such as H5N1 and H7N9 [47–51]. Except for human coronavirus OC43 and HKU1 which might have originated from rodents [52], the rest including human coronavirus 229E, NL63, MERS-CoV, SARS-CoV-1, and SARS-CoV-2 were believed to have jumped from bats to humans via an intermediate host.

It is likely that most interspecies jumping of microbes from animals to humans only results in asymptomatic, symptomatic, or fatal infection without further transmission. Two recent examples are the porcine coronavirus HKU15-related porcine deltacoronavirus which infected Haitian children [53,55] and the canine-feline alphacoronavirus recombinant which infected Malaysian patients [56]. But the MERS-CoV has repeatedly jumped from camels (intermediate host) to humans, and it is often associated with severely symptomatic infection with a fatality rate of 34% [39]. Fortunately, MERS-CoV only occasionally causes person-to-person transmission and rarely large healthcare-related outbreaks [57]. The 2003 SARS-CoV-1 jumping from civets (intermediate host) has caused highly symptomatic disease and large community and healthcare associated outbreaks with a crude fatality rate of 10% [38]. The other end of the spectrum is the 2019 SARS-CoV-2 which is highly transmissible with lots of asymptomatic, pre-symptomatic, or mildly symptomatic infection and a low crude fatality rate of 0.1%–2% [40]. The effective transmission during asymptomatic infection (long incubation periods before onset of disease) such as in the case of HIV may also play a role in sustaining the pandemic for a long period of time.

Based on the above factors, efforts have been implemented to tackle the situation of emerging pandemics. In view of the significant threat of arbovirus due to climate change, the Global Arbovirus Initiative was launched on 31 March 2022 [58,59]. It aims to raise global awareness of the potential epidemic and pandemic risk of arboviruses. It is designed to monitor arboviral diseases under the six pillars: monitor and anticipate risk, reduce epidemic risk, strengthen vector control, prepare and prevent pandemics, enhance innovative approaches, and build a coalition of partners. However, there are potential obstacles to this initiative. The majority of arboviral outbreaks were occurring in developing countries, where poverty may hinder the implementation of public health measures. Furthermore, developing countries may not have the political commitment to this initiative due to other priorities. There is a need for an increase in international efforts to halt and reverse deforestation, so as to protect and restore the ecosystem, and reduce the damage of habitat destruction as well as climate change. Stringent measures should be implemented in reducing bushmeat trafficking and consumption, wildlife trade should be vigorously controlled eventually banned, and warm-blooded vertebrates should not be allowed in markets where farm-level biosecurity measures cannot be possibly enforced.

## Surveillance for emerging agents for the next pandemic

Besides the above measures, more effort on microbial and disease surveillance using both indicator- and event-based approaches should be put into patients with symptomatic disease and sick animals, especially those with severe disease and unexplained death. Massive reduction of population in wild birds or other animals has been followed by zoonotic outbreaks in humans due to *Orthomyxoviridae* [60], *Filoviridae* [61], *Flaviviridae* [62], and *Paramyxoviridae* such as the Nipah virus. Therefore, such a surveillance approach would be more cost-effective in picking up candidates with pandemic potential before their further spread to humans. Specimens collected from symptomatic patients, and sick or dead animals should be first screened by multiplex nucleic acid amplification tests for known pathogens or RNA viruses [63,64], and specimens with a negative screen or from clinically suspicious or inconsistent cases should be further tested by unbiased next-generation sequencing to detect novel zoonotic viruses [65,66]. Although the amount of nucleic acid of unknown viruses is usually low in surveillance specimens, the yield can be increased by the use of Sequence-Independent, Single-Primer-Amplification (SISPA) [67,68]. The combination of a comprehensive database and multiple innovative bioinformatics pipelines can overcome the problems of frequent mutations in RNA viruses and the lack of reference genomes. Another area for surveillance is to focus on the human–animal interface, especially in high-risk locations (such as abattoirs, markets, farms, and clinical or high-containment laboratories). Workers and especially animals with unusual tolerance to viral infections such as bats, pangolins, civets, camelids, and birds [69,70], should be the prime targets for surveillance. Clinical specimens from sick workers and faeces or corpses of the above animals are excellent specimens for microbial surveillance. Dead bird surveillance in Hong Kong has led to the discovery of the fourth coronavirus genus, including sparrow coronavirus HKU17, which is closely related to porcine coronavirus HKU15 and porcine deltacoroanvirus jumping into Haitian children [54]. Wet market surveillance should be performed in a sustained manner instead of performing only in response to outbreaks. Viruses with epidemic and pandemic potential, including avian influenza H5N1 [71], H7N9 [72], SARS-CoV-1 [73], and SARS-CoV-2 [19], have been linked to the markets. A similar surveillance approach should be considered for international travellers with fever or relevant clinical symptoms especially when alerted by an early warning system or soft intelligence of suspected outbreaks in their country of origin due to uncertain agents.

The suspected pathogens identified from next-generation sequencing should be cultured in animal organoids system such as intestinoids followed by testing their degree of pathogenicity in animals. The successfully isolated animal virus could then be tested for interspecies jumping in human organoids such as airway organoids as in the case of bat SARS-CoV-2 [74–77]. Besides knowing their potential for jumping from animals to humans, their degree of viral tropism for upper nasal, middle airway, and lower alveolar organoids could predict the degree of transmissibility and disease severity as illustrated by different variants and subvariants of SARS-CoV-2 [44]. The pathogenicity of these animal viruses which can infect human organoids should then be defined in non-human primates to understand the pattern of the disease in humans. Pathogens with high potential to cross the animal–human species barrier are candidates for the design of rapid diagnostics including polymerase chain reaction and rapid antigen tests, broad-spectrum antivirals, and vaccines. Further studies on the ability of personal protective equipment against the candidate pathogen should also be conducted. More rigorous evaluation is crucial to pre-empt future spillover events and implement appropriate preventive measures to prevent another possible pandemic.

## Early detection of the next pandemic

Early indicators of emerging infectious diseases include abnormally severe diseases in young healthy adults or healthcare workers, and an abnormal increase in sales of over-the-counter drugs or traditional Chinese medicine for fever and flu symptoms. The event-based surveillance system has become more popular in recent years due to its ability in picking up these indicators and its proactive nature in the detection of epidemics. During the COVID-19 pandemic, artificial intelligence (AI) early warning systems became the centre of attention. AI systems incorporate technologies such as text mining and machine learning in order to process and filter vast open-source data without significant intervention from humans to generate early warning signals in pandemic surveillance.

The advantages of AI in early warning systems are multi-fold. It is rapid and real-time because the system generates epidemic alerts through the processing of open-source data online all over the world. It requires minimal human intervention and therefore can address the issue of lack of expertise in the field theoretically. It is also applicable in developing countries as it does not require additional manpower, and has been demonstrated to be able to detect the Ebola epidemic earlier even in areas with relatively low smartphone penetration [78]. It has the capability to combine with other software tools for risk analysis and simulation, in order to forecast the severity of upcoming epidemics so as to prioritize resources. Most of the AI systems are available on the Web free-of-charge so that different sectors can access these tools for early warning and pandemic preparation. Although most of the AI early warning systems, such as Metabiota and Epidemic Intelligence from Open Sources (EIOS), only perform analysis for specific pathogens, other systems, such as HealthMap and EPIWATCH, can perform analysis through a syndrome-based approach, therefore allowing early detection of epidemics even for unknown pathogens.

The major drawback of event-based surveillance is that most of the information processed is non-specific when compared with the indicator-based surveillance approach, unless the information is based on credible sources such as health care professionals, or the information is of particular concern, such as an unexplained severe acute respiratory illness in health care workers or patients with animal contacts. It is labour-intensive for public health officials to verify each signal detected from event-based surveillance and to perform detailed microbial surveillance of each event. Therefore, event-based surveillance alone may not be as useful for catching the emerging agents for the next pandemic. Furthermore, depending on how the AI technology is being regulated, censorship may alter AI algorithms leading to distorted reporting of results.

The AI early warning systems should aim to achieve rapid control of outbreaks. The future direction for the development of AI is the inclusion of multi-dimensional data such as the travel history of individuals, distribution of vectors and animals in different areas, temperature, and humidity for spatiotemporal analysis of future outbreaks. Furthermore, the combination of animal data with spatiotemporal data of infected cases can help to identify important animal–human interfaces that lead to microbial spillover, therefore allowing public health officials to collect surveillance specimens and implement targeted measures to prevent future outbreaks.

However, the outbreaks of 2003 SARS-CoV-1 and 2019 SARS-CoV-2 were initially picked up in the news or social media. Indeed, when a doctor voiced their concern about seeing patients with respiratory diseases of unusual severity on social media in 2019, or when young, healthy, and health-conscious healthcare workers were hospitalized for atypical pneumonia in 2003, these are important soft intelligence worthy of serious attention. Other possible areas that event-based surveillance should target are large international gatherings, which may be opportunities for outbreaks which can then spillover internationally. Examples include *Neisseria meningitidis*, *Vibrio cholerae*, and arboviruses associated with the Hajj pilgrimage, Hepatitis A, and Mpox outbreaks associated with Pride events [79–82]. If the AI early warning systems may help to pick up a useful signal of the next important outbreak, it is likely by the monitoring of fever, respiratory symptoms, sick leave, or hospitalization in healthcare workers, workers at the animal–human interface, and attendees of international gatherings.

Experience from past epidemics due to novel respiratory viruses showed that unidentified or non-typable viruses at laboratories have led to the discovery of human cases caused by the 1997 avian influenza A H5N1 in Hong Kong, the 2003 SARS-CoV-1, the 2009 pandemic influenza A H1N1 in the US, the 2012 MERS-CoV in Saudi Arabia, the 2013 avian influenza A H7N9 and the 2019 SARS-CoV- 2 in Wuhan. As acute community acquired pneumonia is a very common clinical entity leading to hospital admission, these emerging infections were not easily picked up or alerted by the local syndromic surveillance or early warning systems which may imply the low sensitivity of these approaches, but have been picked up by routine clinical laboratory surveillance for isolates not identified by conventional laboratory tests.

## Prevention and preparation for the next pandemic

Another important area for preparation and prevention of the next pandemic is careful regulation of biosafety level 3 and 4 laboratories to ensure both safety and productivity, as the next pandemic can potentially be related to laboratory incidents or bioterrorism [83]. There were numerous examples of laboratory-associated infection leading to subsequent outbreaks. In 1978, an outbreak of smallpox occurred at the University of Birmingham Medical School, United Kingdom; the laboratory was working with variola virus and it is believed that a poorly maintained service duct was the cause of the outbreak [84]. The 1977 Russian influenza A H1N1 which highly resembled a strain circulating globally from 1946 to 1957 was suspected to have leaked accidentally from a laboratory or from a live-vaccine trial [85,86]. Some cases of SARS in 2004 were most likely acquired in the laboratory, resulting in the subsequent spread of the disease in the community and hospital settings [87]. In 2004–2005, samples containing live influenza A H2N2 viruses (A/Japan/305/57) were accidentally sent to different international laboratories by the College of American Pathologists, leading to the immediate recall of the samples to prevent disastrous outcomes [88]. A biopharmaceutical plant in Lanzhou discharged the vaccine strain of *Brucella* species into the waste gas which infected at least 10,000 adjacent residents in 2019 [89].

For the preparation for future epidemics and pandemics, vaccine development is important in the prevention of infection, transmission, and reduction in the severity of disease in case of infection, such as in the case of influenza and COVID-19 [90,91]. Moreover, adequate supply of oxygen, drugs for symptom control (antipyretics) and immunomodulation (steroid) could also play an important role. Vaccines, especially those with mucosal protection, may halt further transmission of the pathogen when given during outbreaks, Therefore, versatile vaccine platforms and sufficient vaccine production facilities which have the surge capacity to produce timely amounts of vaccines or replenish stockpiles may be crucial to control the spread of potential emerging pandemics. An example of a prepared vaccine is the 17DD yellow fever vaccine, which was given in the Yellow Fever outbreak in Angola and the Democratic Republic of Congo to control further spread of yellow fever virus in 2016 [92].

## Difficulties in the developing countries

Performing microbial surveillance in tropical and subtropical regions is valuable, as human–animal interfaces are common in these areas with frequent spillover events. However, there are potential challenges faced by underdeveloped countries including a paucity of expertise to perform regular surveillance, and the lack of public health investment and interest in surveillance when compared with clinical testing and medical countermeasures for tackling pandemics.

Addressing the problem of limited resources in developing countries but at the same time maintaining the balance between enhancing pandemic preparedness and microbial surveillance is difficult. For the hardware of microbial surveillance by sequencing, the COVID-19 pandemic has strengthened the development of regional sequencing centres, especially in developing countries. However, with subsequent variants of SARS-CoV-2 showing a reduction in disease severity, most of the countries are scaling back pandemic control resources, and focusing on the restoration of usual economic activities. Political leaders should be convinced of the value of the investment in improving their laboratories for pandemic preparation, and to equip the laboratories with the ability for high throughput whole genome sequencing. Furthermore, a centralized laboratory at a regional or national level for high throughput sequencing is another solution for these challenges. A centralized laboratory allows focused funding for purchasing sequencing equipment, concentrating expertise in a single location, and at the same time central processing of all surveillance specimens, so as to allow the selection of representative samples in order to utilize resources effectively. In addition, the use of AI early warning systems can bypass the problem of the lack of manpower. Other parties such as the pharmaceutical and biotechnology companies should also help by limiting their price when providing reagents, consumables, medications, and vaccines to developing countries, as these companies may benefit from the data generated in the epidemics occurring in these areas.

## Future pandemics

Emerging RNA viruses from animals have caused various pandemics in human history. SARS-CoV-2 appeared to be another example of zoonotic spillover from animals to humans. Similar to influenza A, the highly transmissible SARS-CoV-2 undoubtedly causes significant morbidity, mortality, and economic loss through disruptions of our daily socioeconomic activities. Though arboviruses are highly confined by the ambient temperature governing the activity of arthropods, genetic mutations, recombination and reassortment can be frequent in *Reoviridae* and *Bunyavirales* with segmented genomes which may allow them to emerge as pandemic agents with worsening climate change [93]. Besides acute explosive respiratory or systemic diseases, we must never forget silent pandemics due to agents like HIV or antimicrobial-resistant bacterial or fungal pathogens. The former will predispose patients to many opportunistic agents while the latter can cause almost untreatable bacterial (Carbapenem-resistant *Enterobacteriaceae*, pan-drug-resistant *Acinetobacter baumannii*) or fungal (*Candida auris*, Mucorales) superinfections in patients with acute respiratory disease [94].

## Conclusion

In summary, the increasing global population fuelling the dietary demand for food animals, the intrusion and exploitation of wildlife habitats leading to more frequent human–animal interactions, the intensive farming practice and poor hygienic conditions in wet markets, the rapidity of microbial dissemination by frequent air travel, the change in the distribution of animals and arthropods due to climate change, and frequent mutations in RNA viruses appear to be important factors driving the emergence of pandemics. More extensive and sustained animal surveillance for monitoring the evolution of novel viruses and risk assessment for zoonotic emergence of another influenza virus, a putative SARS-CoV-3, a bat HKU4/5 related or MERS-CoV related Merbecovirus [95], a lineage D bat coronavirus HKU9 related CoV [96], another novel enterovirus or paramyxovirus such as henipaviruses causing outbreaks or even another pandemic are warranted. Surveillance especially at human–animal interfaces and sick travellers is no less important. Careful regulation of biosafety level 3 and 4 laboratories, as well as the development and stockpiling of broad spectrum antiviral drugs, vaccines, oxygen supply and respiratory support equipments, symptomatic medication (antipyretics, cough mixture), immunomodulatory agents (steroid), and reusable personal protective equipment are also important. Effective electronic tracking device for rapid multilayer contact tracing for case isolation and contact quarantine is no less important than high surge capacity of healthcare manpower, isolation wards, and quarantine facilities. The government should focus more resources on vaccination of the elderly as age is one of the risk factors for severe infection. Further advancement of AI technology is likely one of the future directions for event-based surveillance, but censorship is one important concern. It may help the identification of important human–animal interfaces and hotspots, so as to reduce the risk of microbial spillover. Such preparations may nip the bud of the next pandemic, and minimise the morbidity, mortality and loss of productivity with earlier resumption to normalcy.
